# TomatoGuard-YOLO: a novel efficient tomato disease detection method

**DOI:** 10.3389/fpls.2024.1499278

**Published:** 2025-01-31

**Authors:** Xuewei Wang, Jun Liu

**Affiliations:** Shandong Provincial University Laboratory for Protected Horticulture, Weifang University of Science and Technology, Weifang, China

**Keywords:** tomato disease detection, YOLOv10, multi-path inverted residual unit, dynamic focusing attention framework, focal-EIoU loss function

## Abstract

Tomatoes are highly susceptible to numerous diseases that significantly reduce their yield and quality, posing critical challenges to global food security and sustainable agricultural practices. To address the shortcomings of existing detection methods in accuracy, computational efficiency, and scalability, this study propose TomatoGuard-YOLO, an advanced, lightweight, and highly efficient detection framework based on an improved YOLOv10 architecture. The framework introduces two key innovations: the Multi-Path Inverted Residual Unit (MPIRU), which enhances multi-scale feature extraction and fusion, and the Dynamic Focusing Attention Framework (DFAF), which adaptively focuses on disease-relevant regions, substantially improving detection robustness. Additionally, the incorporation of the Focal-EIoU loss function refines bounding box matching accuracy and mitigates class imbalance. Experimental evaluations on a dedicated tomato disease detection dataset demonstrate that TomatoGuard-YOLO achieves an outstanding mAP50 of 94.23%, an inference speed of 129.64 FPS, and an ultra-compact model size of just 2.65 MB. These results establish TomatoGuard-YOLO as a transformative solution for intelligent plant disease management systems, offering unprecedented advancements in detection accuracy, speed, and model efficiency.

## 1 Introduction

With the advent of artificial intelligence (AI) and the global shift towards smart agriculture, precision farming techniques have emerged as essential tools for addressing crop disease challenges in modern agricultural production (Li et al., 2021). AI has significantly transformed the field of plant disease detection, enabling more accurate and timely diagnosis while becoming an increasingly integral part of agricultural practices, particularly in identifying crop diseases (Wang et al., 2020). Among the most widely cultivated crops globally, tomatoes are highly susceptible to various diseases such as late blight, leaf mold, and bacterial speck. These diseases can cause extensive damage, leading to severe plant wilting and significant reductions in yield. The economic impact of such outbreaks is substantial, underscoring the critical importance of timely detection and control measures to ensure food security and sustain agricultural productivity.

Traditional methods for disease detection often rely on visual diagnosis by experienced agronomists. While effective in certain contexts, these approaches are inherently subjective and prone to inconsistencies (Kaniyassery et al., 2024). Moreover, as agricultural production scales up, manual monitoring of crops becomes increasingly inefficient and imprecise, rendering it inadequate to meet the demands of large-scale farming operations (Singh et al., 2020).

To address these limitations, AI-based image recognition technologies have been increasingly utilized in crop disease identification. In particular, deep learning-based object detection algorithms have demonstrated significant potential in automating disease detection in both drone-assisted and greenhouse environments. This has driven research efforts toward developing efficient, accurate, and scalable automated disease detection systems that can enhance agricultural productivity and resilience against crop diseases.

Object detection, a fundamental research area within computer vision, primarily comprises traditional methods and deep learning-based approaches. Traditional methods, such as SIFT and HOG features paired with SVM for object detection (Lowe, 2004), continue to grapple with limitations in detection accuracy and generalization when confronted with complex backgrounds and varied disease symptoms (Zhang et al., 2018; Liu et al., 2018; Zhu et al., 2019).

The advancement of deep learning technology has significantly propelled the development of CNN-based object detection techniques for tomato disease recognition, encompassing Faster R-CNN (Girshick, 2015), SSD (Liu et al., 2016), and YOLO (Redmon et al., 2016) (Fuentes et al., 2017). Among these, the YOLO series of algorithms has garnered substantial attention owing to its speed and accuracy. With the introduction of YOLOv4 (Bochkovskiy et al., 2020), YOLOv5 (Jocher et al., 2022), YOLOX (Ge et al., 2021), YOLOv6 (Li et al., 2022), YOLOv7 (Wang et al., 2023), and other epoches, performance levels have consistently improved. The continuous evolution of YOLO algorithms demonstrates significant improvements in object detection capabilities through systematic architecture optimization and technical innovations. The core architectural enhancements include implementing more efficient backbone networks for feature extraction, optimizing neck structures for better feature fusion, introducing advanced head designs for more accurate predictions, and incorporating attention mechanisms for improved feature focus. These fundamental improvements are complemented by technical innovations such as enhanced loss functions for better convergence, adaptive feature aggregation for multi-scale detection, improved anchor-free detection mechanisms, and advanced data augmentation strategies.

The latest YOLOv10 algorithm has achieved innovations in multiple aspects, significantly enhancing detection accuracy and speed (Wang et al., 2024; Alif and Hussain, 2024). Specifically, the BGF-YOLOv10 algorithm, designed for small object detection, achieved a remarkable mAP of 42.0% on the VisDroneDET2019 dataset, demonstrating significant improvement over earlier versions (Mei and Zhu, 2024). Additionally, the BLP-YOLOv10 model, optimized for safety helmet detection in low-light environments, achieved an impressive mAP of 98.1% (Du et al., 2024). This model excels in feature extraction and image processing by adjusting backbone channel parameters, incorporating sparse attention mechanisms, and integrating low-frequency enhancement filters.

Nevertheless, YOLOv10 still faces challenges in small object detection, complex background interference, and multi-scale target handling (Kang et al., 2024; Lee et al., 2024; Sangaiah et al., 2024). Compared to other versions, the decision to improve YOLOv10 was based on several key factors. First, YOLOv10 introduces a lightweight architecture and multi-path convolution modules, significantly enhancing adaptability in complex environments while maintaining precision and optimizing computational efficiency, making it suitable for real-time, resource-constrained scenarios. Second, its modular design provides high flexibility for integrating novel mechanisms such as adaptive attention and inverted residual units, making it well-suited for large-scale disease detection tasks. Lastly, YOLOv10 has demonstrated excellent performance in practical applications. For example, BGF-YOLOv10 has improved crop disease detection in agricultural scenarios, while BLP-YOLOv10 has shown exceptional robustness in industrial settings under challenging lighting conditions (Mei and Zhu, 2024; Du et al., 2024). These comprehensive improvements and remaining challenges make YOLOv10 an ideal candidate for continued development and optimization, particularly in addressing specific application requirements while maintaining general-purpose detection capabilities. The balance between addressing current limitations and leveraging existing strengths positions YOLOv10 as a promising framework for future advancements in object detection technology.

To significantly enhance the accuracy and efficiency of tomato disease object detection, this study proposes an improved algorithm based on YOLOv10. The research framework comprises three key stages. First, a self-built tomato disease dataset is constructed to provide high-quality input data, including standardized annotation and preprocessing. Second, during model construction, the proposed Multi-Path Inverted Residual Unit (MPIRU) and Dynamic Focusing Attention Framework (DFAF) enhance feature extraction and small target detection capabilities. Additionally, the optimization of the loss function and class balance strategies improves overall detection performance. Finally, the effectiveness of the enhanced model is validated through comparative experiments with existing models. This study aims to provide robust technical support for early detection and precise control of tomato diseases, contributing to the development of intelligent agriculture and ensuring food safety and agricultural production efficiency.

## 2 Literature review

### 2.1 Agricultural disease detection: from traditional methods to deep learning

In recent years, artificial intelligence and deep learning technologies have been gradually applied to various agricultural domains, such as crop cultivation, harvesting, and disease detection (Kamilaris and Prenafeta-Boldú, 2018). Farmers have begun using smartphones to detect crop diseases and pests (Liu et al., 2020). However, this field still faces numerous challenges and development opportunities.

Traditional agricultural disease detection and identification methods primarily rely on manual feature extraction. While these methods have achieved certain research results (Camargo and Smith, 2009; Shekhawat and Sinha, 2020), they have apparent limitations. These conventional approaches necessitate substantial professional expertise and a deep reservoir of knowledge, are inherently subjective, and often neglect valuable attributes that are challenging to identify with unaided human perception. Furthermore, when confronted with voluminous data in authentic natural settings, the precision of traditional methods frequently deteriorates significantly (Buja et al., 2021; Wiesner-Hanks et al., 2019).

In contrast, deep learning technologies, with their potent feature representation capacities, can autonomously extract features from voluminous multi-type disease data, thereby substantially enhancing detection performance (Saleem et al., 2021; Bhattacharya et al., 2022). AI has made disease detection more automated, efficient, precise, and reliable, primarily manifested in the following aspects:

1. Automatic feature learning: Capable of automatically learning and recognizing disease features from millions of images, greatly enhancing detection accuracy.
2. No manual intervention: Does not require manual feature extraction or threshold setting, can automatically adapt to various environments and conditions.
3. Efficient processing: Capable of rapidly identifying a substantial quantity of diseases, thereby enhancing detection efficiency and accuracy.

**Figure 1:**
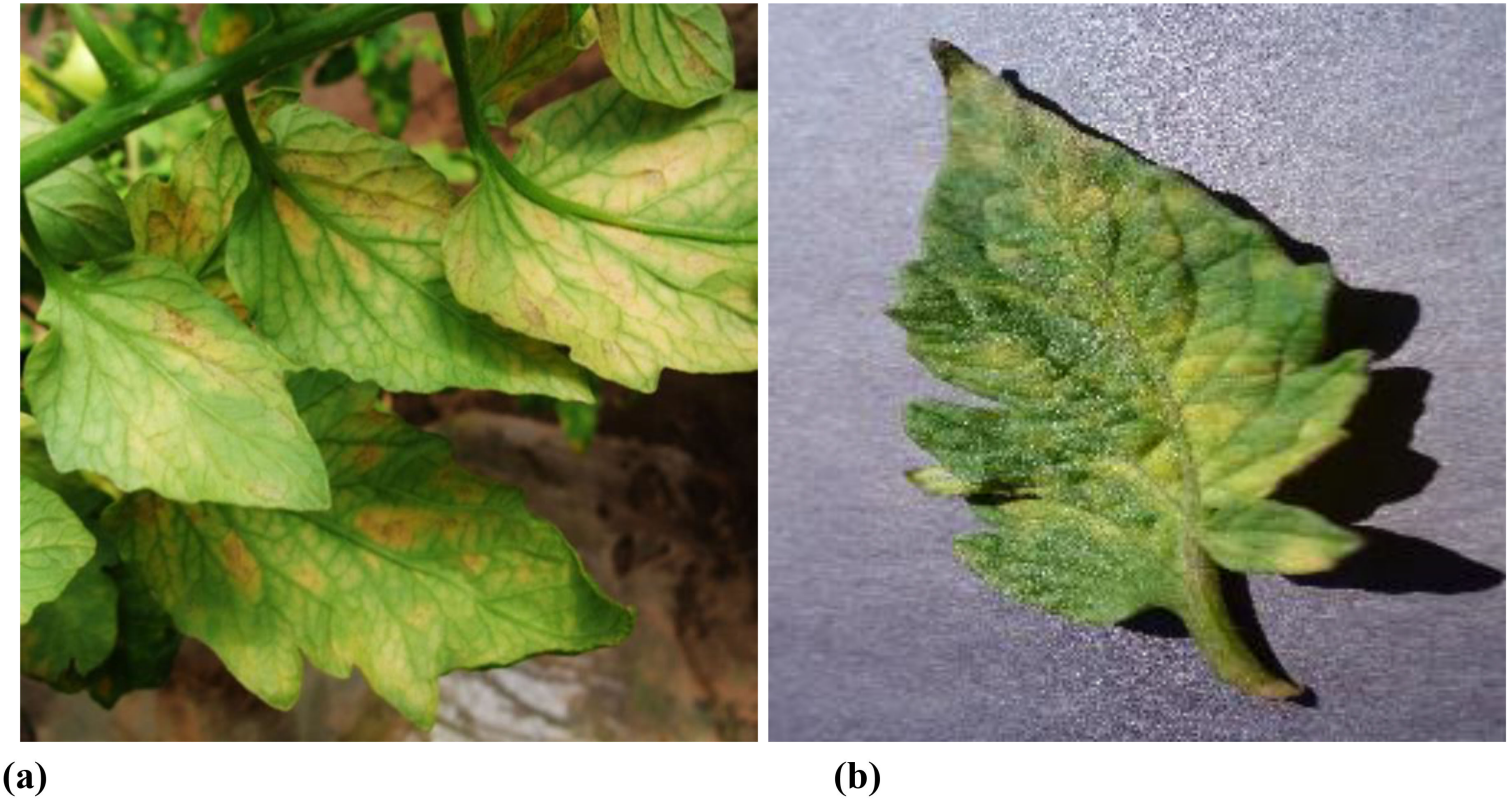
Dataset examples. **(A)** natural environment photography (with background) **(B)** ideal environment (without background).

The efficacy of deep learning models in plant disease detection is intrinsically tied to the quality of the training data. Currently, the creation of datasets for agricultural disease detection models can be broadly classified into two primary categories: natural environment photography (with background) and ideal environment (without background), as depicted in **Figure 1**. Images captured in natural environments present complex backgrounds, which can enhance the robustness and generalization capabilities of trained models. Conversely, images acquired in ideal environments lack background distractions, but the resulting models often struggle to achieve satisfactory detection performance in real-world scenarios.

### 2.2 Agricultural disease detection in ideal environments: achievements and limitations

Given the intricate relationship between agricultural diseases and factors like cultivation practices, management strategies, and climate fluctuations, prevailing open-source tomato disease datasets largely depend on laboratory samples, exemplified by AI Challenger 2018, Kaggle, and PlantVillage. Recognizing the considerable time and effort necessary to accumulate a substantial quantity of natural environment samples, numerous agricultural disease detection model studies predominantly leverage open-source ideal environment samples for training purposes. Although models developed based on ideal environment samples have achieved notable outcomes in laboratory settings, many have not undergone validation in natural environments. Existing research suggests that these models are primarily well-suited for scenarios where disease-affected areas constitute a significant portion of the image but may encounter difficulties in handling complex backgrounds, lighting variations, changes in shooting angles, and diverse lesion sizes within natural scenes.

For instance, Bora et al. (2023) developed a system capable of detecting diseases on tomato leaves, stems, fruits, and roots with remarkable accuracy rates of 99.84%, 95.2%, 96.8%, and 93.6%, respectively. Zhang et al. (2023) introduced the M-AORANet model, which demonstrated exceptional recognition accuracy of 96.47% on a dataset comprising 3,123 tomato leaf images. Sunil et al. (2023) employed a Multi-level Feature Fusion Network (MFFN) to achieve an impressive external test accuracy of 99.83% on publicly available tomato disease datasets. Although these models exhibited exceptional performance in controlled environments, their primary limitation lies in their inability to pinpoint the exact location of lesions within images, hindering their direct application in real-world agricultural settings.

### 2.3 Agricultural disease detection in natural environments: progress and challenges

Although models trained on natural environment data more accurately reflect real-world conditions, the majority of research continues to concentrate on model development, refinement, and structural analysis using personal computers. This neglects the critical need for lightweight and highly accurate solutions in practical applications.

Several prior studies have explored the use of deep learning for plant disease detection in various agricultural settings. Li et al. (2019) developed a mobile application for early detection of tomato late blight, demonstrating the potential for smartphone-based disease diagnosis. Sun et al. (2021) proposed the MEAN-SSD model, achieving an accuracy of 83.12% for apple leaf disease detection while maintaining real-time processing speeds (12.53 frames per second). Zhang K. et al. (2021) incorporated skip connections within the Faster R-CNN architecture, achieving an accuracy of 83.34% on a custom dataset of soybean disease images. Similarly, Chen et al. (2021) constructed a model for cucumber leaf disease detection with an accuracy of 85.52%. Finally, Dananjayan et al. (2022) demonstrated the effectiveness of YOLOv4 for rapid and accurate detection of citrus leaf diseases. Qi et al. (2022) introduced an enhanced SE-YOLOv5s network model that achieved an impressive 91.07% accuracy on the tomato disease test set. While these studies demonstrated real-time disease recognition capabilities, the models developed for individual agricultural diseases face challenges in widespread deployment due to the fluctuating nature of disease occurrences.

Machine vision detection of tomato diseases faces significant obstacles in actual planting environments, including complex growing conditions, multiple disease types, and subtle symptom variations (Barbedo, 2018; Kadry, 2021; Fuentes et al., 2021), which impose exceptionally high demands on the multi-feature and cross-scale extraction capabilities of detection algorithms. While the YOLO series models have garnered widespread adoption for their swift and precise detection capabilities, there remains potential for enhancement in feature extraction and detection accuracy within complex environments.

Considering the current research status and challenges, this study proposes a tomato disease detection method based on a refined YOLOv10 architecture. By meticulously analyzing tomato disease types and image characteristics, the algorithm is iteratively enhanced and experimentally validated, aiming to fulfill the accuracy and speed requirements of intelligent tomato disease detection and reduce manual diagnosis costs. The innovations of this study are primarily as follows:

1. The introduction of the Multi-Path Inverted Residual Unit (MPIRU) significantly enhances the model’s ability to fuse multi-scale features through parallel processing across multiple paths, effectively reducing the number of model parameters.
2. Integration of a Dynamic Focusing Attention Framework (DFAF) into the C2f module, improving the focus on important target areas and localization accuracy.
3. By incorporating Focal-EIoU as a refined loss function, we significantly enhanced the model’s ability to accurately match objects while effectively mitigating the challenges posed by imbalanced datasets.
4. The improved YOLOv10 performs excellently in detection accuracy, parameter optimization, and complexity control, making it suitable not only for tomato disease detection but also for other crop disease detection tasks in complex backgrounds.

## 3 Materials and methods

### 3.1 Data collection and dataset preparation

In this study, we utilized a custom-built tomato disease dataset aimed at capturing disease features in real agricultural environments, reflecting the imbalanced nature of disease occurrence in actual scenarios. The data collection process was rigorously controlled to ensure quality and representativeness. **Table 1** summarizes the key parameters of data collection.

**Table 1:** Overview of data collection parameters.

Parameter	Description
Collection equipment	Agricultural IoT monitoring equipment (HS-CQAI-1080)
Location	Tomato production base, Shouguang City, Shandong Province, China
Precise coordinates	Longitude: 118.782956°, Latitude: 36.930686°
Image resolution	3648 × 2056 pixels
Daily collection times	08:30–11:30 and 14:30–17:30
Distance between device and lesions	0.2~0.5 meters
Environmental conditions	Sunny and cloudy days; various infected regions and conditions
Total number of images	10,537

**Figure 2:**
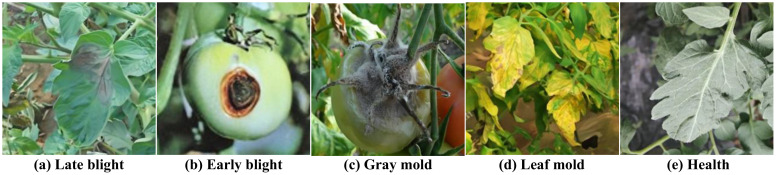
Shows five samples of the various tomato diseases in the dataset: **(A)** Late blight **(B)** Early blight **(C)** Gray mold **(D)** Leaf mold **(E)** Health.

The data acquisition process prioritized capturing a broad spectrum of environmental conditions, encompassing diverse lighting scenarios, angles of view, and background elements, such as leaves, weeds, and soil. This diversity is crucial for improving the model’s generalization ability and applicability in real-world conditions. Each image was accompanied by rich metadata, including environmental temperature, precise location, and timestamp, providing valuable context for subsequent analysis and model training. **Figure 2** shows representative samples of the various tomato diseases in the dataset, visually illustrating the characteristics and complexity of different diseases.

Our dataset reflects the natural distribution of disease occurrence in actual agricultural environments, thus exhibiting significant class imbalance. **Table 2** details the sample count for each category and its proportion in the overall dataset.

**Table 2:** Sample count for each category of our dataset.

Category	Sample count	Proportion
Healthy	3526	33.46%
Late Blight	2745	26.05%
Early Blight	2103	19.96%
Gray Mold	1358	12.89%
Leaf Mold	805	7.64%
Total	10,537	100%

This imbalanced distribution reflects the relative frequency of various diseases in actual agricultural production, providing us with a realistic challenge scenario. In particular, the sample sizes for leaf mold and gray mold are notably smaller than other categories, highlighting the importance and difficulty of identifying rare diseases in practical applications.

To address the dataset’s class imbalance, we employed a stratified random sampling technique. This method ensured that the distribution of each category in the training, validation, and testing sets accurately mirrored the original dataset. A detailed breakdown of the dataset division is provided in **Table 3**.

**Table 3:** Dataset division.

Dataset	Sample size	Proportion
Training set	8430	80%
Validation set	1054	10%
Test set	1053	10%
Total	10,537	100%

This division method ensures that each subset contains balanced representations of all categories while preserving the imbalanced characteristics of the original dataset, contributing to stable performance and reliable evaluation of the model in real-world scenarios.

### 3.2 Data annotation

**Figure 3:**
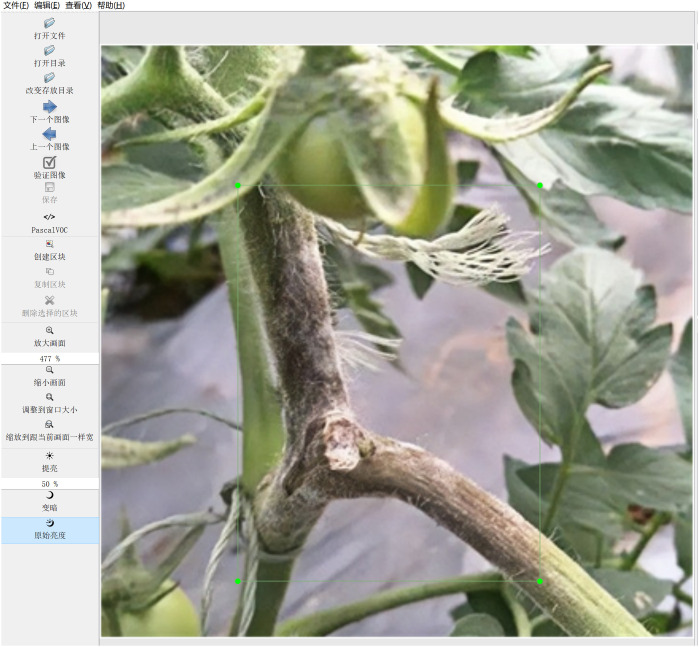
Data Annotation Process. Using LabelImg tool for disease area annotation. Example of generated VOC format XML file.

Accurate data annotation is indispensable for guaranteeing the efficacy of model training. This research employed the widely utilized open-source annotation tool Labellmg to prepare data for object detection purposes. The annotation process is visually depicted in **Figure 3**.

### 3.3 Data augmentation

Given the dataset’s uneven distribution, we implemented focused data augmentation techniques designed to address class imbalance concerns and bolster the model’s capacity to identify uncommon classes.

#### 3.3.1 Offline data augmentation

To improve model generalization and mitigate overfitting, this study performed data augmentation on the training set. When selecting data augmentation methods, we paid special attention to preserving key disease features while avoiding unnecessary distortions. We adopted more aggressive offline data augmentation strategies for categories with fewer samples (such as leaf mold and gray mold). **Table 4** outlines the offline augmentation methods and their parameters applied to different categories.

**Table 4:** Offline data augmentation methods (for different categories).

Category	Augmentation Methods	Parameters
Leaf mold	Horizontal flip, Vertical flip, Small angle rotation, Brightness adjustment	Rotation range: ± 20°, Brightness range: ± 15%
Gray mold	Horizontal flip, Vertical flip, Small angle rotation	Rotation range: ± 15°, Brightness range: ± 10%
Early blight, Late blight	Horizontal flip, Vertical flip	–
Healthy samples	Horizontal flip	–

**Figure 4:**
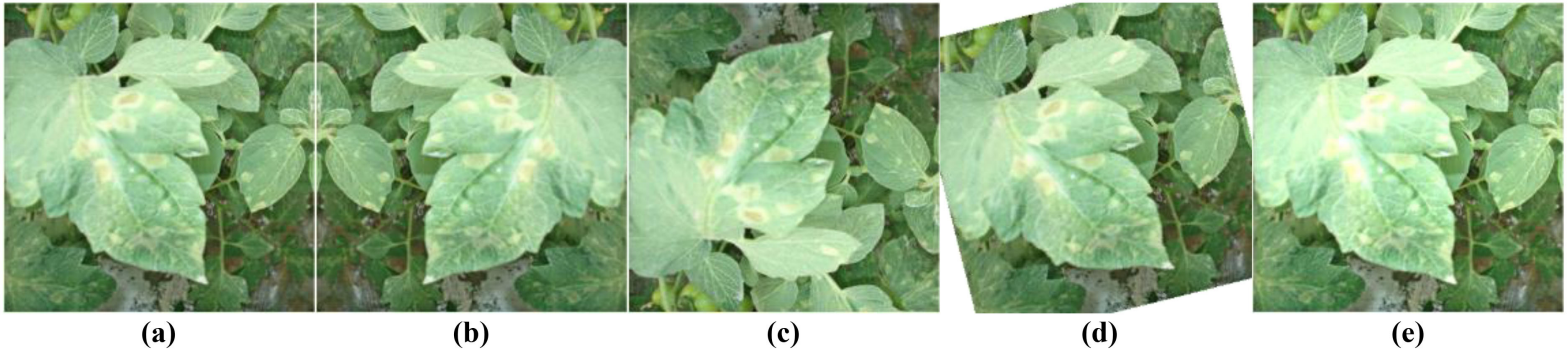
Offline data augmentation examples. **(A)** Original image; **(B)** Horizontal flip; **(C)** Vertical flip; **(D)** Small angle rotation; **(E)** Brightness adjustment.

**Figure 4** demonstrates the effects of four image processing techniques. Through this strategy, we significantly increased the sample size of rare categories while maintaining the overall diversity of the dataset. This approach helps balance the performance across categories, especially improving the model’s ability to recognize relatively rare diseases such as leaf mold and gray mold.

Through the offline data augmentation strategy, we significantly expanded the total number of samples. The original dataset contained 10,537 images, which increased to 31,053 after augmentation, approximately 2.95 times the original size. Notably, for rare categories such as leaf mold and gray mold, the sample counts increased from 805 and 1358 to 4025 and 5432, respectively. This category-specific augmentation approach effectively mitigated the class imbalance issue, improved the model’s ability to recognize rare categories, and preserved critical disease features, thereby providing a more robust foundation for the model’s generalization performance.

#### 3.3.2 Real-time data augmentation

Given the dataset’s uneven distribution and the intricate agricultural setting, we implemented a variety of real-time data augmentation techniques within YOLOv10 with the objective of enhancing the model’s capacity for generalization and its aptitude for identifying uncommon categories (**Table 5**).

**Table 5:** Real-time data augmentation methods.

Method	Parameters	Purpose
Mosaic	Probability = 1.0, Number of images = 4	Increase contextual information, improve small object detection performance
Random Affine	Rotation = ± 10°, Scale = 0.8~1.2	Simulate different shooting angles and distances
MixUp	Probability = 0.15	Increase sample diversity, improve model generalization ability
Random HSV	Hue = ± 10, Saturation = 0.5, Value = 0.5	Simulate different lighting and weather conditions
Cutout	Probability = 0.3	Improve model robustness to partial occlusions

This all-encompassing real-time data augmentation approach not only substantially expands the diversity of the training dataset but also enhances the model’s capacity to adapt to a wide range of intricate scenarios. Especially for disease categories with fewer samples (such as leaf mold and gray mold), these enhancement methods help the model learn more diverse feature representations from limited samples. Through this approach, we expect the model to better handle the complex and variable real-world scenarios in agricultural production, improving detection accuracy for various diseases, particularly under challenging conditions such as insufficient lighting, partial occlusion, or unfavorable shooting angles.

### 3.4 The improved tomato disease detection model based on YOLOv10

Although the C2f module in YOLOv10 enhances feature extraction capabilities through the Bottleneck structure, its equal treatment of all channels and positional information introduces a significant amount of irrelevant interference. This results in suboptimal performance when handling multi-scale small targets and complex backgrounds in tomato disease images. Additionally, in the YOLOv10 model, the backbone network extracts deep features through multiple down-sampling convolution layers.

**Figure 5:**
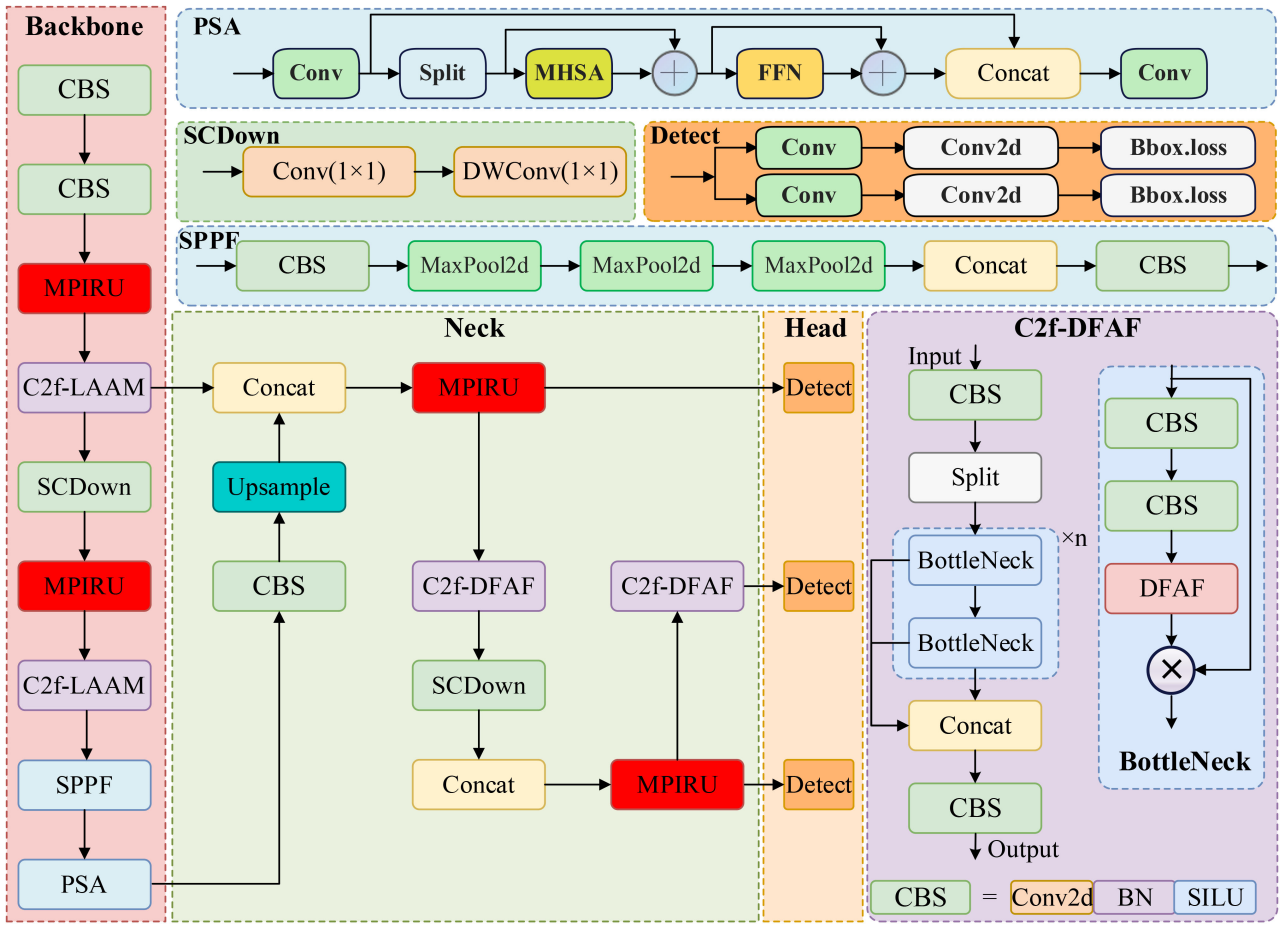
The architecture of the proposed TomatoGuard-YOLO model.

Although this multi-stage downsampling enhances the model’s capacity to handle large targets and intricate backgrounds, it also leads to a significant reduction in small target features. To mitigate the loss of these crucial details during detection, the neck network utilizes multiple upsampling operations to restore feature map resolution and integrate features from various levels, thereby improving the model’s ability to detect targets of varying sizes. However, this alternating process of downsampling and upsampling also results in excessive layer stacking within the backbone and neck networks, increasing the model’s parameter count and computational complexity, making it challenging to meet real-time detection requirements in practical applications. To overcome these aforementioned challenges, given the nature of multi-scale target detection of tomato diseases in intricate environments, this study proposes a streamlined target detection algorithm, TomatoGuard-YOLO, as illustrated in **Figure 5**.

This model is an enhancement of version n of YOLOv10 (referred to as YOLOv10 unless otherwise specified), featuring lightweight designs for both the backbone and neck networks. Initially, a novel feature extraction and fusion module, termed the Multi-Path Inverted Residual Unit (MPIRU), is devised. Subsequently, the proposed Dynamic Focusing Attention Framework (DFAF) is integrated into the C2f module, resulting in the C2f-DFAF module. Subsequently, all C2f modules within the foundational YOLOv10 backbone and neck networks are supplanted with MCIR and C2f-DFAF. Furthermore, the backbone network’s downsampling operations have been curtailed to retain a greater quantity of feature information. Concurrently, the upsampling and feature concatenation procedures within the neck network have been streamlined to diminish the number of layers and complexity, thereby further reducing computational expenses. In conclusion, the loss function has been meticulously refined to Focal-EIoU, effectively rectifying the deficiencies of the original loss function and bolstering the model’s capacity to concentrate on a variety of samples.

The subsequent sections elucidate each improved module, aiming to elevate the model’s performance in detecting tomato diseases in intricate agricultural environments, particularly in handling small targets, complex backgrounds, and class imbalance challenges.

#### 3.4.1 Multi-path inverted residual unit

**Figure 6:**
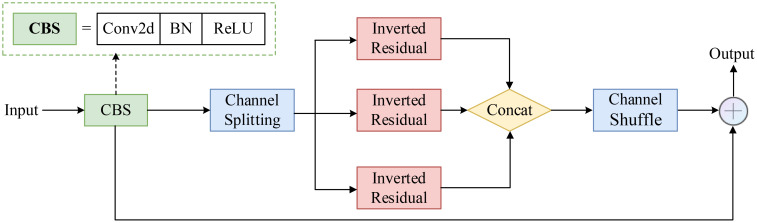
Structure of the Multi-Path Inverted Residual Unit (MPIRU).

In complex agricultural environments, tomato diseases often exhibit multi-scale and multi-form characteristics. To bolster the model’s capacity to extract these intricate features, we introduce the Multi-Path Inverted Residual Unit (MPIRU). The design of MPIRU incorporates the inverted residual structure from MobileNetV2 (Sandler et al., 2018) and the channel separation concept from ShuffleNetV2 (Ma et al., 2018), aiming to enhance feature extraction diversity while preserving computational efficiency. **Figure 6** illustrates the detailed structure of MPIRU.

As shown in **Figure 6**, MPIRU first evenly divides the input feature map (X) into (n) branches {X_1_, X_2_,…, X_n_}, with each branch independently processing a portion of the channels. Each branch adopts an “expand-convolve-squeeze” inverted residual structure, which can be expressed as:


(1) $$F_{i}\left(X_{i}\right)=H_{i}\left(G_{i}\left(E_{i}\left(X_{i}\right)\right)\right)$$

where *E_i_*, *G_i_*, and *H_i_* represent the expansion (1x1 convolution), depthwise separable convolution (3x3), and compression (1x1 convolution) operations, respectively. The output (Y) of MPIRU can be expressed as:


(2) $$Y=Concat\left(F_{1}\left(X_{1}\right),F_{2}\left(X_{2}\right),\cdots ,F_{n}\left(X_{n}\right)+X\right)$$

Where Concat denotes the concatenation operation along the channel dimension.

To facilitate information exchange between different branches, we apply a channel shuffle operation after concatenation. This design not only enhances the diversity of feature extraction but also maintains relative computational stability.

#### 3.4.2 C2f-DFAF module

Accurately locating and identifying diseased areas is essential in disease detection tasks. Attention mechanisms have proven highly effective in enhancing the architecture of deep neural networks, achieving notable success in various applications. However, their integration into lightweight networks has significantly lagged behind their implementation in larger models. This disparity arises primarily because most mobile and edge devices have limited computational resources, making it challenging to accommodate the high overhead associated with traditional attention mechanisms. To address this limitation, we propose the Dynamic Focusing Attention Framework (DFAF), which is seamlessly integrated with the C2f module to form the novel C2f-DFAF module, delivering efficient and effective attention capabilities suitable for lightweight networks.

This enhancement is inspired by SENet (Hu et al., 2018) and CBAM (Woo et al., 2018). SENet employs 2D global pooling to calculate channel attention and improves model performance with a relatively minimal computational overhead. Nevertheless, SENet solely considers inter-channel information and neglects positional information, which is essential for capturing object structures in visual tasks. To address this limitation, CBAM endeavors to compute positional information by reducing the channel dimension of the input tensor and subsequently utilizing convolution to calculate spatial attention. However, convolution solely captures local area information and cannot model long-distance dependencies, nor can it capture spatial information at varying scales to enrich the feature space. Although CBAM uses two fully connected layers and a nonlinear Sigmoid function to generate channel weights, capturing nonlinear cross-channel interaction information and controlling model complexity through dimensionality reduction, the parameter count is still positively correlated with the square of the input feature map channels. Further research shows that dimensionality reduction negatively impacts channel attention prediction, with low efficiency in capturing dependencies among all channels.

**Figure 7:**
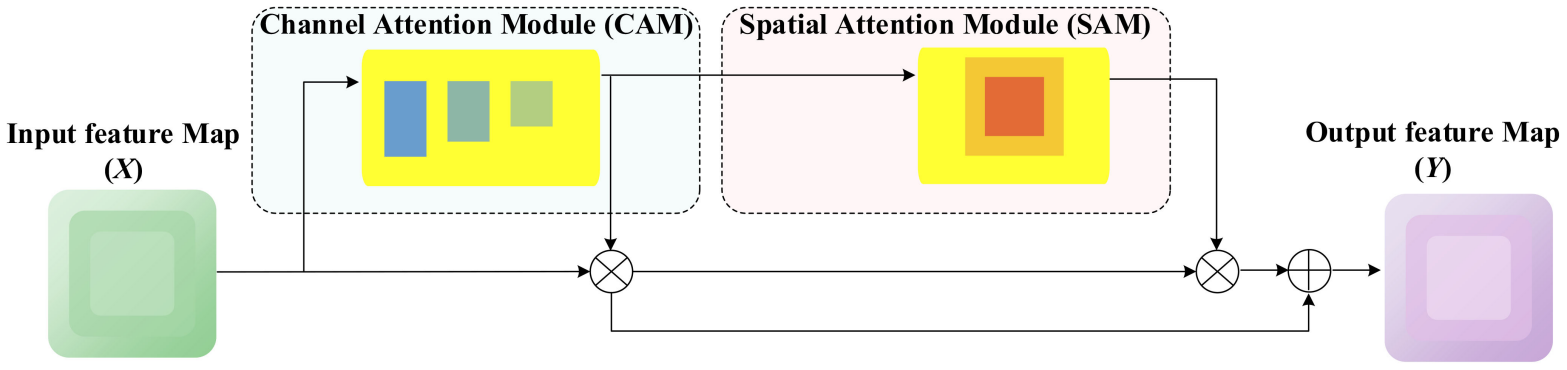
Structure of DFAF attention mechanism.

To address the critical challenges in object detection, we introduce the C2f-DFAF module, centered around a lightweight adaptive attention unit (DFAF) capable of dynamically learning and adjusting the significance of features across channel and spatial dimensions. DFAF effectively pinpoints tomato disease features, suppresses irrelevant features, and significantly reduces parameters and computational overhead. The module comprises a feature input layer, channel attention module layer, spatial attention module layer, and feature output layer. **Figure 7** illustrates the detailed structure of the DFAF module.

The DFAF (Dynamic Feature Adaptive Fusion) module consists of two primary components: a channel attention module and a spatial attention module. The input features undergo parallel processing through these modules, with their outputs being adaptively fused through multiplication operations. A skip connection preserves the original feature information, ensuring robust feature representation. This architecture enables dynamic feature weighting while maintaining computational efficiency. Specifically, the feature input layer is responsible for receiving and pre-processing raw input features, preparing them for subsequent attention mechanism modules. The channel attention module layer learns weights for each channel, adaptively enhancing important feature channels while suppressing secondary channels, thereby modeling the global importance of features. The spatial attention module layer focuses on capturing spatial dependencies within feature maps by generating attention weight maps, precisely localizing tomato disease regions. Finally, the feature output layer integrates the outputs of the aforementioned attention mechanisms, generating more focused and discriminative feature representations.

The mathematical expression of the DFAF module is as follows:


(3) $$A=\sigma \;\left(W_{C}⊗AvgPool\left(X\right)+W_{S}⊗MaxPool\left(X\right)\right)$$

In the aforementioned formular, *X* represents the input feature, $W_{C}$ and $W_{S}$ are the weight parameters for channel and spatial attention, respectively, σ is the sigmoid activation function, and ⊗ denotes the convolution operation. This design allows the model to adaptively balance the significance of channel and spatial attention, thereby better accommodating different types of disease features.

Simultaneously, the C2f-DFAF module introduces a residual learning mechanism. As shown in Equation 4, the output of the attention mechanism is added to the original features rather than simply multiplied:


(4) Y=X*A+X

This design helps mitigate the vanishing gradient problem while preserving the original feature information, which is particularly important for maintaining the subtle features of diseases. Consequently, the DFAF module utilizes the channel attention module to generate channel-level attention maps, thereby enhancing the response to small targets. The spatial attention module generates spatial-level attention maps through convolution operations, enabling the model to accurately pinpoint regions of interest in intricate backgrounds. By integrating these two attention mechanisms, the model can adjust and weight channel and spatial attention, allowing it to concentrate on significant regions in complex backgrounds, strengthening small target detection capabilities, and improving recognition accuracy. Additionally, the use of global pooling and convolution operations enables efficient parallel computation without adding too many extra parameters, allowing the C2f-DFAF module to achieve excellent detection performance with high efficiency and low parameters in tomato disease detection.

#### 3.4.3 Focal-EIoU loss function

Tomato disease samples in images often exhibit substantial class imbalance, with significant variations in shape and size. To address these challenges, we propose the Focal-EIoU loss function, which integrates the sample balancing capability of Focal Loss (Lin et al., 2017) and the precise bounding box regression capability of EIoU (Efficient IoU) (Zhang Z. et al., 2021).

Focal Loss is particularly effective in addressing class imbalance issues by dynamically reducing the loss weight of easily classified samples, thereby increasing the model’s emphasis on difficult-to-classify samples. The definition of Focal Loss is as follows:


(5) $$FL\left(p_{t}\right)=-\alpha_{t}{\left(1-p_{t}\right)}^{\gamma }\log\left(p_{t}\right)$$

(6) $$\alpha_{t}=\frac{v}{\left(1-IoU\right)+v}$$

(7) $$\nu =\frac{4}{\pi^{2}}{\left(\text{arctan}\frac{\omega^{gt}}{h^{gt}}-\text{arctan}\frac{\omega }{h}\right)}^{2}$$

**Figure 8:**
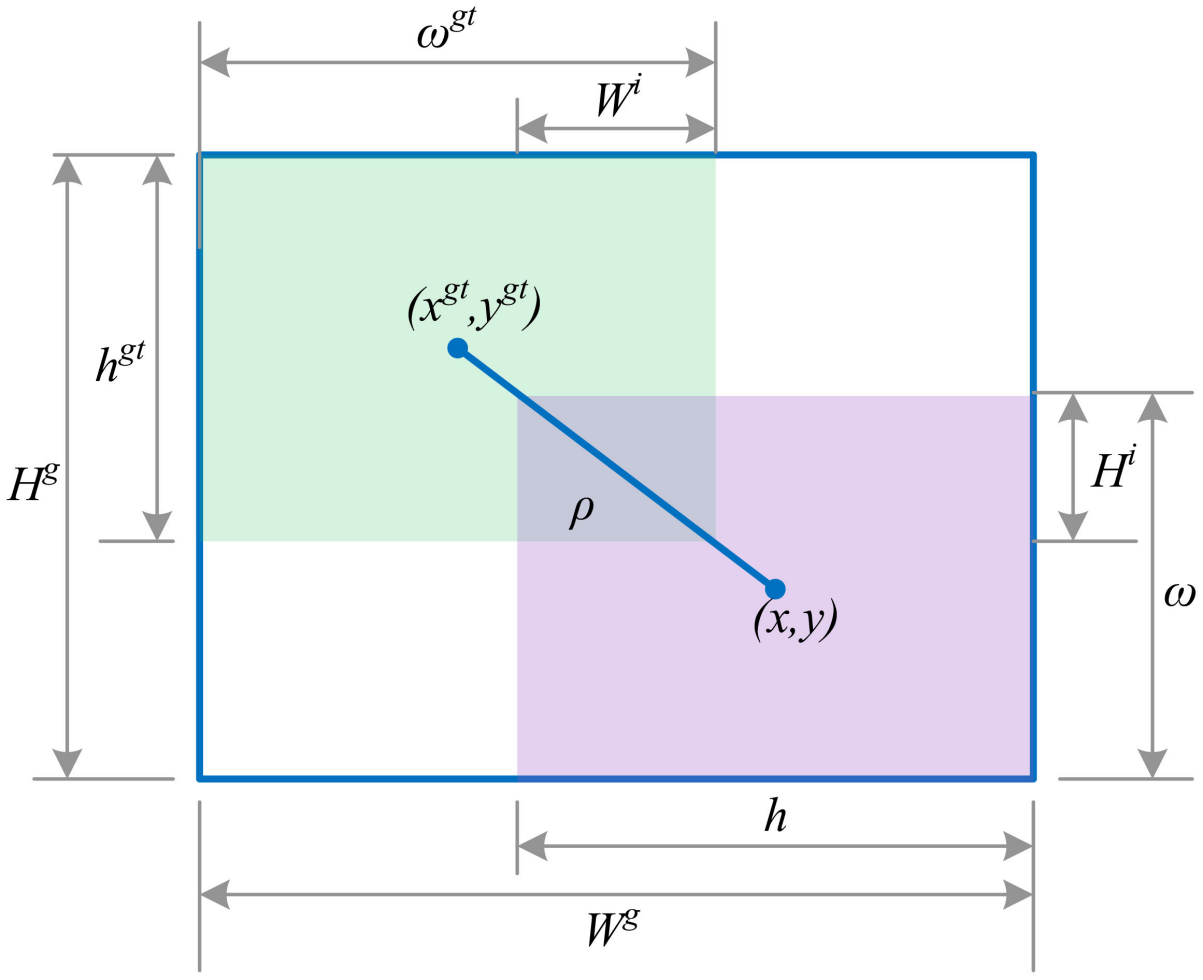
Loss function border diagram.

The model’s predicted probability for the accurate classification is denoted by $p_{t}$ . To address the issue of imbalanced classes, we employ a weighting factor represented by $\alpha_{t}$ . Additionally, the parameter γ serves to regulate the influence of easily classified samples. The dimensions of the ground truth and predicted bounding boxes are expressed as ( $\omega^{gt}$ , $h^{gt}$ ) and ( ω , *h*), respectively. The central coordinates of the predicted and ground truth boxes are given by *b* and $b^{gt}$ . The Euclidean distance between these center points is calculated as *ρ*. The diagonal length of the smallest bounding box encompassing both boxes is represented by *c*. The weighting function is defined as *α*, and the squared disparity in the diagonal angles of the ground truth and predicted boxes is denoted by *v*, as illustrated in **Figure 8**.

Focal Loss effectively prioritizes challenging samples with low intersection over union (IoU) by assigning them greater weights. This mechanism empowers the model to concentrate on more elusive classes, which is indispensable for the identification of uncommon diseases such as leaf mold and gray mold.

EIoU significantly elevates the accuracy of bounding box regression by refining the precision of the overlap between the predicted and ground truth boxes. EIoU comprehensively evaluates not only the area of overlap but also the relative positions, dimensions, and contours of the boxes. Its formal definition is as follows:


(6) $$EIoU=IoU-\frac{\rho^{2}\left(b,b^{gt}\right)}{c^{2}}-\frac{\nu }{c}$$

EIoU provides a more comprehensive evaluation of bounding boxes than traditional IoU by penalizing deviations in position and aspect ratio, improving the localization accuracy for small targets and irregularly shaped diseases.

The final Focal-EIoU loss function integrates Focal Loss and EIoU, enabling the model to effectively mitigate class imbalance concerns and refine the accuracy of bounding box localization. Its mathematical formulation is as follows:


(7) $$L_{Focal-EIoU}=FL\left(p_{t}\right)+\lambda EIoU$$

Where λ is the weighting factor for balancing classification and localization losses. After multiple experiments, the parameter γ=0.9 was selected. The incorporation of Focal-EIoU loss into the TomatoGuard-YOLO model enables it to effectively prioritize less prevalent disease categories and accurately pinpoint the affected regions within intricate agricultural settings.

## 4 Results

### 4.1 Experimental environment

To ensure the reproducibility and reliability of the experimental results, this study meticulously records the experimental environment and key parameter settings. **Tables 6**, **7** list the hardware and software configurations, as well as the core parameters for model training.

**Table 6:** Experimental environment configuration.

Category	Component	Specification
Hardware	Processor	2 × Intel Xeon Platinum 8280 (28 cores, 2.70 GHz)
	Memory	768 GB DDR4-2933 ECC
	GPU	4 × NVIDIA A100 (80 GB HBM2e)
	Storage	2 TB NVMe SSD + 20 TB HDD (RAID 5)
Software	CUDA	CUDA 11.4.2, cuDNN 8.2.4
	Python	Python 3.9.7
	Framework	PyTorch 1.10.1
	Libraries	NumPy 1.21.4, OpenCV 4.5.4, Albumentations 1.1.0

**Table 7:** Model training parameters.

Parameter	Value
Batch Size	64
Initial Learning Rate	0.001
Weight Decay	0.05
Number of Epochs	300

This experiment employs cutting-edge hardware and software configurations, particularly the NVIDIA A100 GPU, whose powerful computing capabilities significantly enhance the efficiency of large-scale model training. The deep learning framework used is PyTorch, a popular open-source platform that seamlessly integrates with the hardware to optimize performance. Additionally, the selected versions of CUDA and cuDNN ensure full utilization of GPU acceleration and provide robust support for neural networks.

These parameters were chosen based on multiple experiments and optimizations. A batch size of 64 effectively utilizes the parallel capabilities of multi-GPU setups. The AdamW optimizer, combined with the cosine annealing strategy, enhances model convergence and mitigates potential overfitting issues during training. To ensure thorough training, an early stopping mechanism is introduced to prevent overfitting on the validation set.

### 4.2 Evaluating indicators

This study employs a set of indicators, such as mAP, Model Size (in MB), Number of Parameters (in MB), and Frames Per Second (FPS). These indicators not only reflect the model’s detection accuracy but also provide insights into its computational complexity and real-time performance. The formulas for these indicators are as follows:


(8) $$Precision=\frac{TP}{TP+FP}\cdot 100\%$$

(9) $$Recall=\frac{TP}{TP+FN}\cdot 100\%$$

(10) $$mAP=\frac{\sum_{i=1}^{K}AP_{i}}{K}$$

(11) $$\;F1-score=\frac{2\cdot Precision\cdot Recall}{Precision+Recall}$$

Where *TP* represents True Positives, *FP* represents False Positives, and *FN* represents False Negatives. Precision measures the proportion of true positive samples among all samples predicted as positive, reflecting the accuracy of the model’s positive predictions. Recall, on the other hand, indicates the proportion of true positive samples correctly identified by the model among all actual positive samples, assessing the model’s recognition ability. The mean Average Precision (mAP) is calculated by averaging the Average Precision (AP) for each class, with *K* denoting the total number of classes. It serves as a comprehensive performance metric in object detection tasks, demonstrating the model’s effectiveness across multiple categories. The F1-score, which is the harmonic mean of Precision and Recall, offers a balanced evaluation of these metrics, particularly useful when there’s an imbalance between them.

In addition to these accuracy evaluation metrics, this study incorporates the following indicators to assess model efficiency:

- **Model Size and Number of Parameters**: Measured in MB, these metrics reflect the model’s complexity and storage requirements. In practical applications, the model’s size and parameter count directly influence memory usage and hardware demands, making them crucial for evaluation.
- **Frames Per Second (FPS)**: This metric indicates the number of image frames the model can process per second during operation. A higher FPS signifies greater operational efficiency, which is essential for real-time detection tasks. FPS not only gauges the model’s performance on hardware but also its potential for real-time applications in various scenarios.

Through a comprehensive evaluation of these indicators, this study not only confirms the accuracy and generalization capabilities of the TomatoGuard-YOLO model in tomato disease detection but also examines its computational efficiency and resource consumption, providing robust support for its practical application.

### 4.3 Learning rate selection

**Figure 9:**
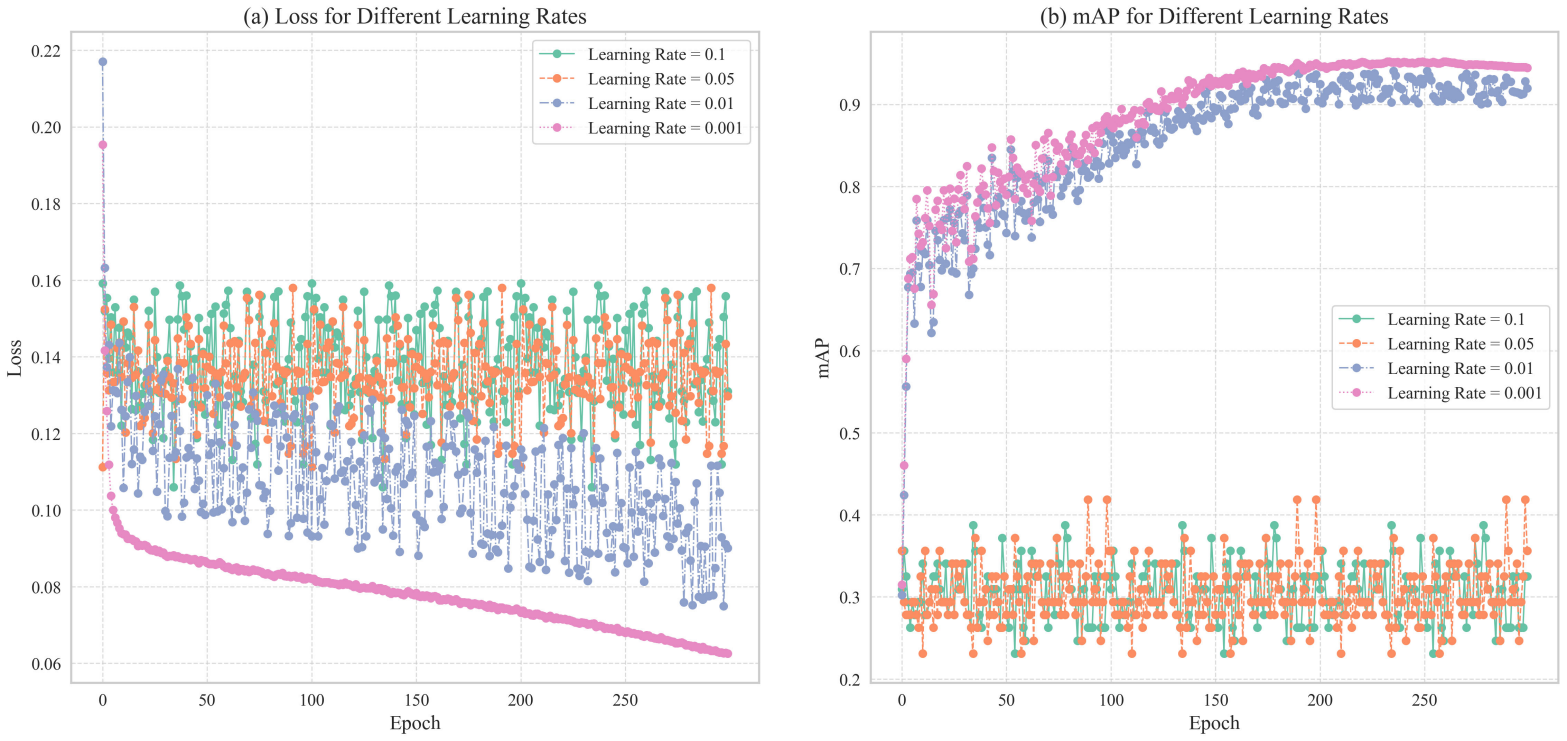
Shows Loss and mAP changes of TomatoGuard-YOLO model under different learning rates: **(A)** Loss for Different Learning Rates **(B)** mAP for Different Learning Rates.

The learning rate is a crucial hyperparameter in training deep learning models, significantly influencing convergence speed, final performance, and stability. To identify the optimal learning rate for the TomatoGuard-YOLO model, we designed multiple comparative experiments, testing initial rates of 0.1, 0.05, 0.01, and 0.001 while keeping other hyperparameters constant. **Figure 9** illustrates the trends in the loss function and accuracy for these different learning rates.

As illustrated in the loss function curves in **Figure 9A**, it is clear that with learning rates of 0.1 and 0.05, the loss function does not exhibit substantial reduction, instead displaying a relatively stable or even fluctuating trend. This suggests that the learning rate is excessively high, resulting in overly large gradient update steps that hinder the model’s convergence to an optimal solution, thereby affecting training outcomes. Conversely, with learning rates of 0.01 and 0.001, the loss function demonstrates a stable and marked downward trend, indicating that the model gradually approaches the optimal solution. Among these, a learning rate of 0.001 yields the most consistent decrease in the loss function, ultimately reaching its lowest point and demonstrating the best convergence.

**Figure 9B** presents the accuracy changes of the model under different learning rates. It is evident that at learning rates of 0.1 and 0.05, the accuracy curves exhibit significant fluctuations and fail to stabilize at a high level. This aligns with the earlier observation of the loss function’s instability, further confirming that a high learning rate induces training volatility, preventing the model from achieving optimal performance. In contrast, at a learning rate of 0.01, the accuracy rises rapidly, but in the mid-to-late training stages, the accuracy curve begins to display slight fluctuations and tendencies towards overfitting. When employing a learning rate of 0.001, accuracy improves steadily, ultimately reaching the highest value without significant overfitting, resulting in a stable training process with excellent convergence.

Considering the convergence behavior of the loss function, the rate of accuracy improvement, and the model’s stability during training, we ultimately selected 0.001 as the initial learning rate for the TomatoGuard-YOLO model. This learning rate ensures stable convergence while providing sufficient capacity for the model to fully adapt to the complex patterns and variations in the training data, laying a solid foundation for subsequent optimizations.

### 4.4 Model training

**Figure 10:**
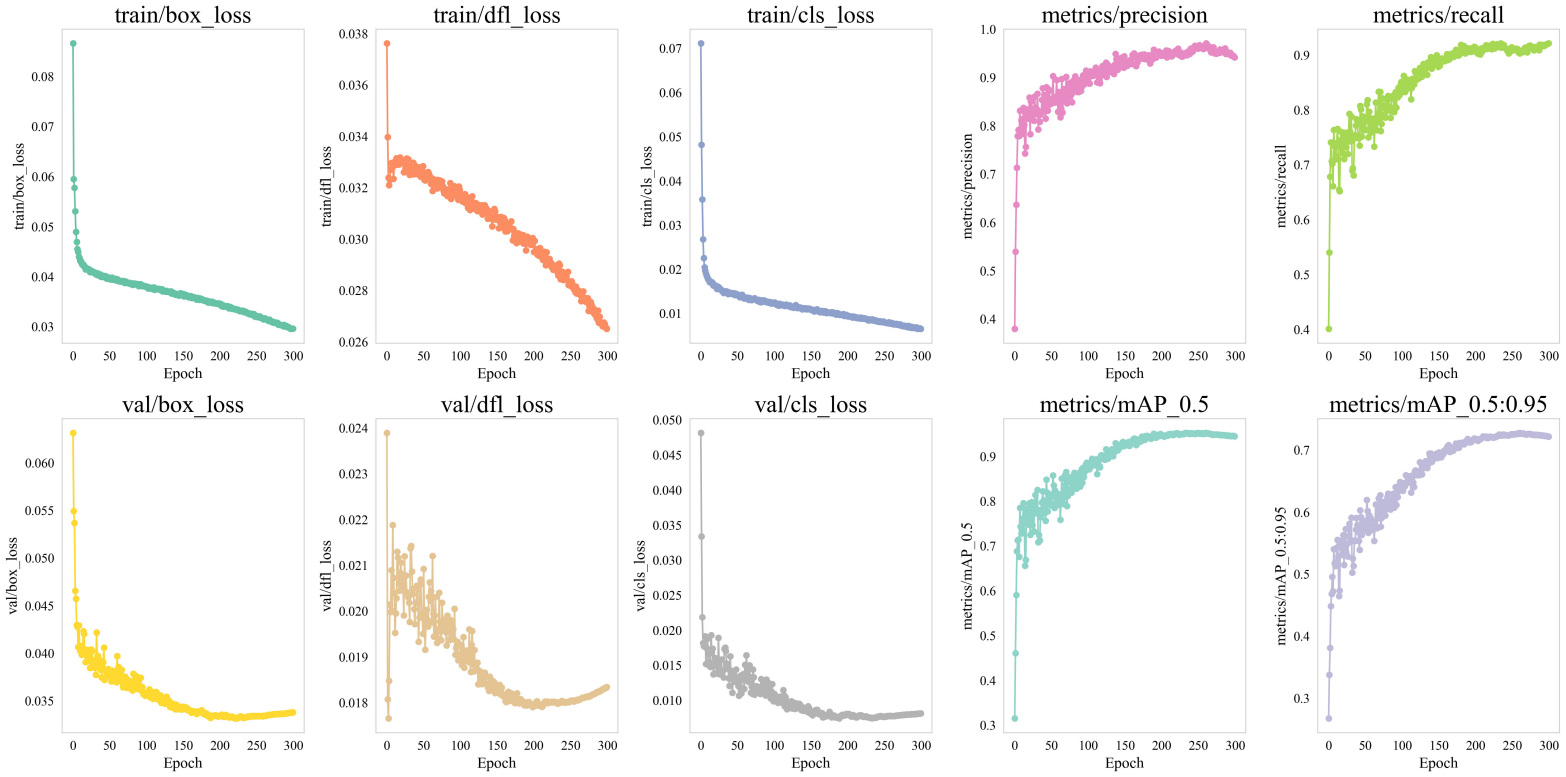
Training process curves.

Based on the experimental environment configuration and parameter settings described earlier, we systematically trained the TomatoGuard-YOLO model for a total of 300 epochs. To comprehensively understand the performance during training, we closely monitored the changes in several key performance indicators, such as the loss function, mAP50, and mAP50:95. **Figure 10** shows the trends of these indicators during the training process.

As illustrated in **Figure 10**, both the training loss and validation loss exhibit a consistent and marked decline, with the gap between them gradually narrowing. This suggests that the model is effectively adapting to the training data while simultaneously enhancing its generalization capability. Specifically, the box_loss decreases from an initial value of 0.086 to around 0.030, obj_loss drops from 0.038 to approximately 0.027, and cls_loss significantly reduces from an initial 0.071 to 0.007. These results indicate notable enhancements in the model’s localization accuracy (box_loss), detection confidence (obj_loss), and classification performance (cls_loss).

In terms of detection accuracy, the mAP50 and mAP50:95 indicators show a rapid increase in the early training stages, followed by a gradual slowdown and eventual stabilization in the later phases. Ultimately, mAP50 reaches 94.23%, while mAP50:95 stabilizes at 72.52%, indicating that the TomatoGuard-YOLO model possesses excellent object detection capabilities across different IoU thresholds.

Additionally, during the training process, we observed that the model’s ability to recognize various types of tomato diseases gradually improved. Notably, it maintained high detection accuracy even for disease types with relatively fewer samples. Through gradual adjustments and optimizations, the TomatoGuard-YOLO model achieved outstanding performance in tomato disease detection.

### 4.5 Ablation study results

To systematically evaluate the effectiveness of our proposed improvements, we conducted comprehensive ablation experiments on the core enhancement modules based on the YOLOv10 model. Through eight carefully designed comparative experiments with different module combinations, we assessed the impact of the MPIRU module, c2f-DFAF module, and Focal-EIoU loss function on tomato disease detection performance across multiple dimensions, including detection accuracy, model size, and computational overhead. **Table 8** presents detailed key performance indicators under various module combinations.

**Table 8:** Ablation experiment results.

Model	MPIRU	c2f-DFAF	Focal-EIoU	mAP50/%	mAP50:95/%	Model Size/MB	Parameters/MB
1				84.15	61.22	5.61	2.73
2	√			91.82	68.71	2.62	0.93
3		√		88.92	65.81	5.62	2.74
4			√	88.72	65.61	5.61	2.73
5		√	√	91.53	68.42	5.62	2.74
6	√		√	93.91	71.23	2.63	0.93
7	√	√		93.37	70.69	2.64	0.94
8	√	√	√	94.23	72.52	2.65	0.94

The experimental results reveal the significant impact of each enhancement module on model performance. The standalone introduction of the MPIRU module led to substantial improvements in detection accuracy, with mAP50 increasing from 84.15% to 91.82% and mAP50:95 rising from 61.22% to 68.71%. More importantly, this performance enhancement was accompanied by a significant reduction in model complexity, with model size decreasing from 5.61MB to 2.62MB and parameter count reducing from 2.73MB to 0.93MB. These results convincingly demonstrate the MPIRU module’s effectiveness in simultaneously improving detection accuracy and achieving model lightweighting.

The integration of the c2f-DFAF module, through optimization of the C2f structure, further enhanced model performance, achieving an mAP50 of 88.92% and mAP50:95 of 65.81%. While the parameter count increased slightly (from 2.73MB to 2.74MB), the significant performance improvements fully justify this optimization. The module demonstrated excellence in enhancing feature hierarchy capture and multi-scale feature fusion, effectively improving the model’s feature expression capabilities.

The application of the Focal-EIoU loss function exhibited unique advantages, improving mAP50 to 88.72% and mAP50:95 to 65.61% without increasing model complexity. This enhancement played a crucial role in optimizing bounding box regression precision and addressing sample imbalance issues while improving the model’s detection robustness across different target scales.

The synergistic combination of all three enhancement modules (Group 8) achieved optimal performance, with mAP50 reaching 94.23% and mAP50:95 rising to 72.52%, while maintaining a compact model size (2.65MB) and parameter count (0.94MB). Compared to the original YOLOv10 model, detection accuracy improved by 10.07 percentage points, while model size and parameter count reduced by 52.76% and 65.57%, respectively. These results conclusively demonstrate the synergistic effects of the enhancement modules in achieving both high-precision detection and model lightweighting objectives.

The ablation study results deeply reveal the core functions of each module: the MPIRU module serves as the foundation for lightweight design, significantly reducing model complexity; the c2f-DFAF module enhances multi-scale target detection capabilities through feature layer optimization; and the Focal-EIoU loss function optimizes detection accuracy while maintaining low computational overhead. The organic integration of these modules enables TomatoGuard-YOLO to achieve lightweight design while maintaining high performance, providing reliable technical support for resource-constrained practical agricultural application scenarios.

### 4.6 Comparative experiments

In the comparative experiments, we standardized the hyperparameters across all models to ensure a fair comparison. Specifically, all models were trained with the following hyperparameters: a batch size of 64, an initial learning rate of 0.001, the AdamW optimizer, and a cosine annealing learning rate scheduling strategy. Training was conducted for 300 epochs with an early stopping mechanism to prevent overfitting. Additionally, to maintain comparability, all experiments were performed on the same hardware environment (NVIDIA A100 GPU), which maximized the performance of each model while ensuring consistent experimental conditions. To assess the efficacy of the TomatoGuard-YOLO algorithm, we performed comprehensive comparative experiments alongside other state-of-the-art object detection algorithms. The results are summarized in **Table 9**.

**Table 9:** Comparative experiment results.

Model	mAP50/%	Parameters/MB	Model Size/MB	FPS
SSD	63.92	24.16	28.42	89.83
FasterR-CNN	70.78	136.75	89.64	21.74
YOLOv3	79.23	61.57	123.65	45.26
YOLOv3-tiny	56.52	8.68	17.53	104.15
YOLOv5s	72.73	7.06	14.52	98.67
YOLOX-s	72.62	9.02	8.95	97.28
YOLOv7-tiny	75.43	6.04	12.32	106.56
YOLOv8n	73.42	3.02	6.22	119.45
YOLOv10	84.15	2.73	5.61	125.62
TomatoGuard-YOLO	94.23	0.94	2.65	129.64

The comparative results presented in **Table 9** clearly demonstrate that the TomatoGuard-YOLO model achieves a significant advantage in the mAP50 metric, reaching 94.23%, which is far superior to other compared algorithms. Especially compared to the original YOLOv10, TomatoGuard-YOLO improves by 10.07 percentage points, fully demonstrating the efficacy of our improvement strategies.

As for model complexity, TomatoGuard-YOLO’s parameter count is only 0.94M, a reduction of 65.57% compared to YOLOv10’s 2.73M. The model size is 2.65M, a reduction of 52.76% compared to YOLOv10’s 5.61M. This indicates that while improving detection accuracy, TomatoGuard-YOLO significantly reduces model complexity, making it a better choice for deployment in resource-limited environments.

Regarding inference speed, TomatoGuard-YOLO achieves an average frame rate of 129.64 FPS, an improvement of 3.20% compared to YOLOv10’s 125.62 FPS. This further demonstrates that despite the significant performance improvements, the model does not sacrifice inference speed; instead, it enhances inference efficiency. This high-efficiency and lightweight characteristic makes TomatoGuard-YOLO perform exceptionally well.

**Figure 11:**
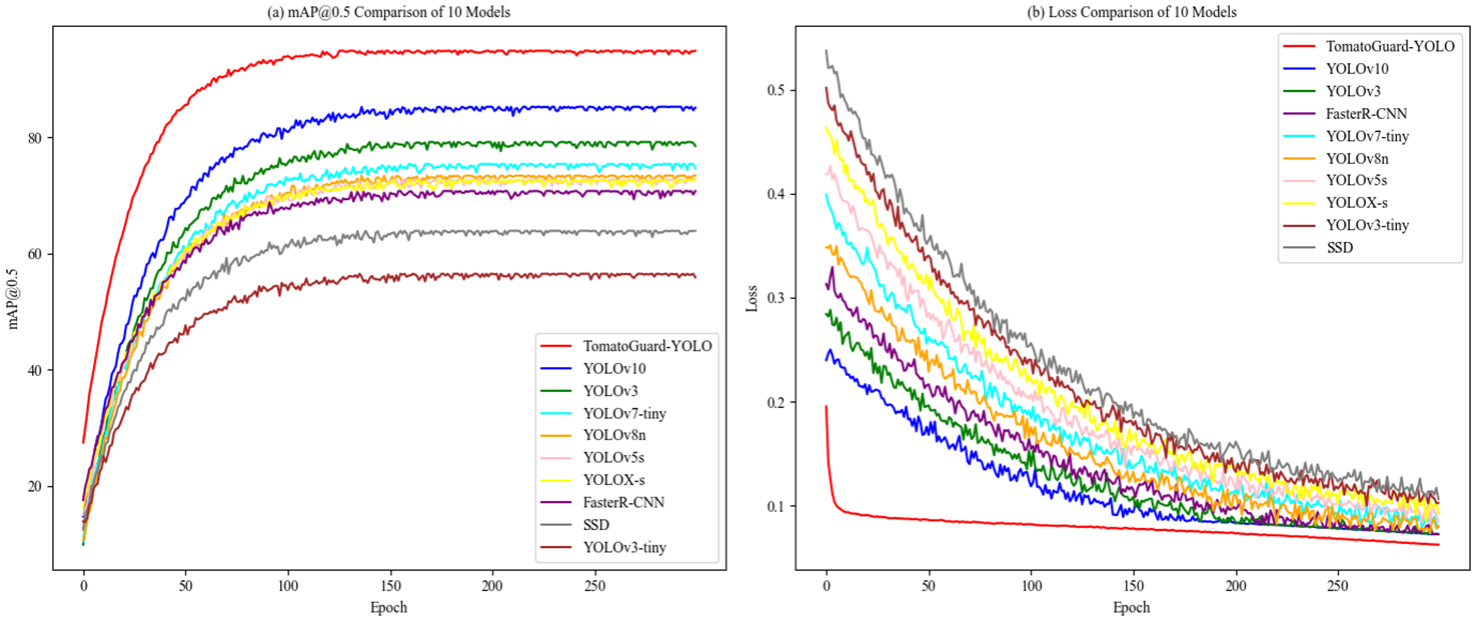
Shows Comparison of mAP and loss curves for different models: **(A)** mAP0.5 Comparison of 10 Models **(B)** Loss Comparison of 10 Models.

To further assess the benefits of TomatoGuard-YOLO, we performed a comparison of mAP and Loss curves with other models, as shown in **Figures 11A, B**.

From **Figure 11A**, the mAP curve for TomatoGuard-YOLO is notably superior to those of the other models under comparison, ultimately stabilizing at approximately 94.23%, which reinforces the model’s strong performance in tomato disease detection. **Figure 11B** illustrates that the initial loss for TomatoGuard-YOLO is 0.195359, significantly lower than that of the other models, eventually stabilizing at around 0.0626034. This suggests that the model achieves a quicker convergence and a lower final loss, further confirming its optimization efficacy. Overall, the outstanding performance of TomatoGuard-YOLO regarding accuracy, speed, and compact design highlights its significant potential for application in tomato disease detection within complex environments.

### 4.7 Detection results for different types of diseases

To thoroughly assess the TomatoGuard-YOLO model’s effectiveness in identifying different tomato diseases, we evaluated it using a custom imbalanced dataset. The detection results for various disease types, as achieved by the TomatoGuard-YOLO model, are summarized in **Table 10**.

**Table 10:** Detection performance of the TomatoGuard-YOLO model across various disease types.

Category	Precision (%)	Recall (%)	F1-score (%)	AP50 (%)
Healthy	96.85	96.53	96.69	97.59
Late Blight	95.93	95.41	95.67	95.99
Early Blight	94.72	94.28	94.51	94.11
Gray Mold	93.16	92.58	92.87	93.07
Leaf Mold	91.74	90.86	91.32	90.36
Average	94.48	93.93	94.21	94.23

**Table 10** demonstrates that the TomatoGuard-YOLO model attains notable Precision, Recall, and AP metrics above 90% for all four disease types and healthy samples, demonstrating high accuracy and recall rates. The model’s mAP reaches 94.23%, fully proving its excellent performance in handling different types of tomato diseases. Notably, the model excels not only in detecting common diseases such as late blight and early blight but also maintains high detection sensitivity for relatively rare diseases like gray mold and leaf mold. Additionally, the model’s outstanding performance in recognizing healthy samples helps reduce misdiagnosis and unnecessary treatments, which is crucial for practical agricultural production.

The AP50 values for detecting five types of tomato diseases and healthy samples using the proposed TomatoGuard-YOLO algorithm and other models are displayed in **Table 11**. The findings clearly demonstrate that the TomatoGuard-YOLO algorithm shows enhanced adaptability in handling targets with pronounced sample imbalance and size variations.

**Table 11:** Comparison of AP50 results for different models in tomato disease detection tasks.

Method	mAP50/%	AP50/%				
		Healthy	Late Blight	Early Blight	Gray Mold	Leaf Mold
SSD	66.56	71.52	69.73	65.84	62.82	62.89
FasterR-CNN	73.56	79.04	77.07	72.77	69.44	69.51
YOLOv3	82.32	88.45	86.24	81.43	77.70	77.78
YOLOv3-tiny	58.68	63.05	61.47	58.04	55.38	55.44
YOLOv5s	76.19	81.87	79.82	75.37	71.92	71.99
YOLOX-s	75.32	80.93	78.90	74.50	71.09	71.16
YOLOv7-tiny	78.82	84.69	82.57	77.97	74.40	74.47
YOLOv8n	83.20	89.39	87.16	82.30	78.53	78.61
YOLOv10	84.15	90.71	87.79	83.72	81.37	77.18
TomatoGuard-YOLO	94.23	97.59	95.99	94.11	93.07	90.36

As shown in **Table 11**, the YOLOv10 algorithm achieves an AP50 of 90.71% for detecting healthy samples but only 77.18% for identifying leaf mold. Similarly, the YOLOv8n algorithm performs relatively well for late blight detection, with an AP50 of 87.16%, but struggles with gray mold, achieving only 78.53%. In contrast, the proposed TomatoGuard-YOLO algorithm demonstrates exceptional performance across all categories, significantly surpassing other models in the overall detection of five types of tomato diseases as well as healthy samples. Notably, for the more challenging cases of gray mold and leaf mold, TomatoGuard-YOLO achieves AP50 values of 93.07% and 90.36%, respectively, outperforming competing algorithms by a substantial margin.

Accurate identification and localization of tomato diseases are critical metrics for evaluating detection performance. While healthy samples and late blight are relatively easier to detect due to their distinct features, diseases such as early blight, gray mold, and leaf mold pose greater challenges. These diseases often exhibit subtle symptoms, with blurred boundaries between lesions and healthy tissue, making detection more difficult. Gray mold and leaf mold, in particular, are characterized by irregular lesion distribution, significant variations in lesion size, and high visual similarity to the background, further complicating accurate detection.

Compared to the next-best performer, YOLOv10, TomatoGuard-YOLO achieves a remarkable improvement of 10.08 percentage points in mAP50, with category-specific gains ranging from 6.88 to 13.18 percentage points. This comprehensive performance enhancement highlights the superiority of TomatoGuard-YOLO in tackling the complexities of tomato disease detection. Furthermore, it provides reliable technical support for precise diagnosis and timely intervention, offering significant practical value in real-world agricultural applications.

### 4.8 Visualization of disease detection results

**Figure 12:**
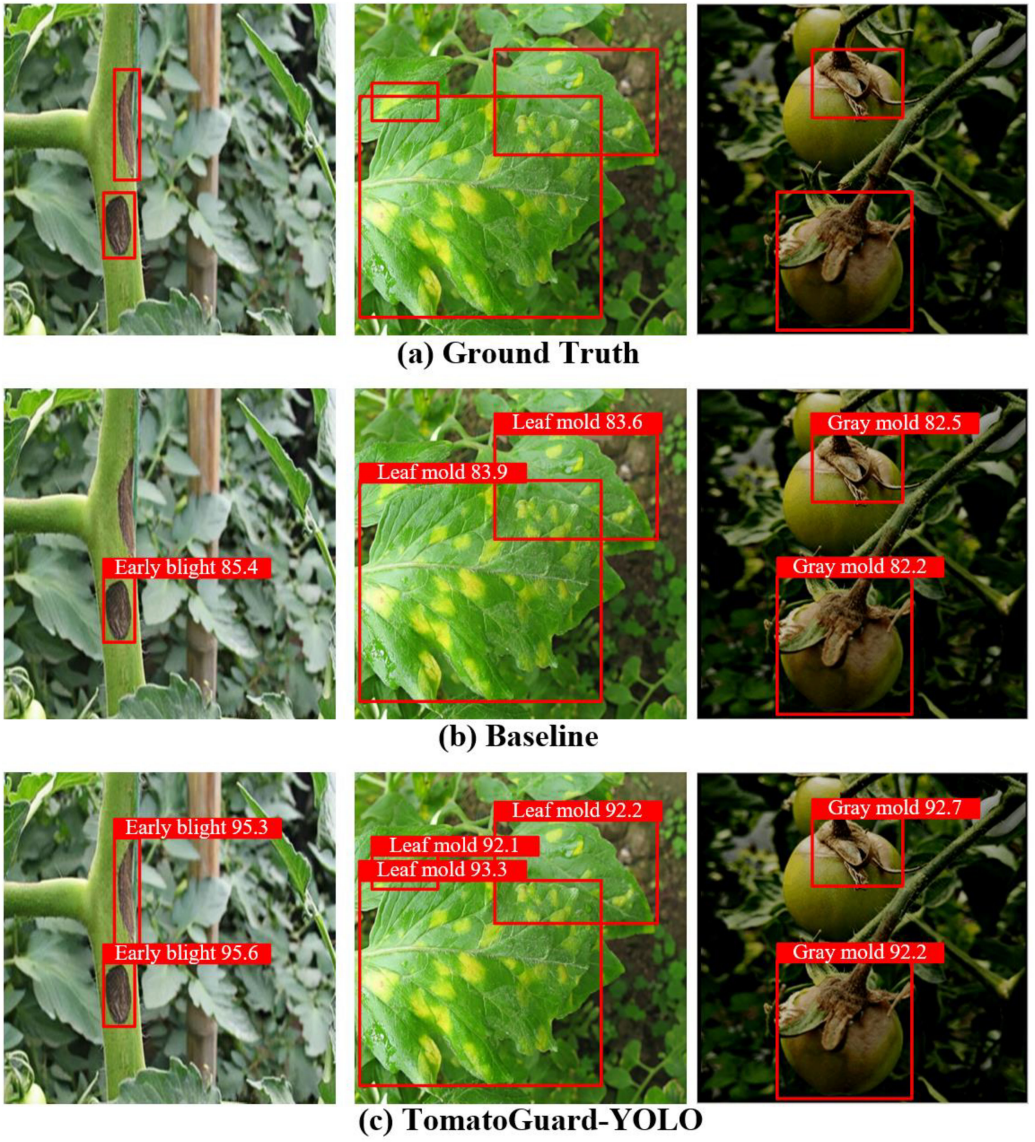
Detection results comparison in typical Tomato disease scenarios **(A)** Ground Truth, **(B)** Baseline, and **(C)** TomatoGuard-YOLO.

To visually compare the detection capabilities of the TomatoGuard-YOLO model, **Figure 12** highlights the performance differences between YOLOv10 and TomatoGuard-YOLO in representative tomato disease scenarios.

The visual results clearly demonstrate that TomatoGuard-YOLO outperforms YOLOv10, particularly in handling complex backgrounds and multi-scale targets. These advantages underscore the robustness and precision of TomatoGuard-YOLO in accurately identifying and localizing diseased regions under challenging conditions, further validating its superiority in practical agricultural applications. In detecting small targets, TomatoGuard-YOLO demonstrates extremely high precision, especially in identifying early disease spots, accurately locating lesions. This is crucial for timely detection and control measures. In complex situations such as overlapping and occluded leaves, TomatoGuard-YOLO can still effectively distinguish disease areas, significantly reducing false detection rates, showing high robustness in complex scenarios. Additionally, in uneven lighting or complex shadow backgrounds, TomatoGuard-YOLO exhibits excellent environmental adaptability, accurately identifying disease areas, ensuring detection stability under different lighting conditions. Compared to YOLOv10, TomatoGuard-YOLO generates more precise bounding boxes, aiding in accurately assessing the severity and spread of diseases, providing more reliable support for actual disease assessment. Therefore, by introducing innovations such as MPIRU, DFAF, and Focal-EIoU loss functions, TomatoGuard-YOLO significantly improves detection accuracy and robustness while maintaining model lightweight, excelling in multi-scale and complex scenarios of tomato disease detection. Moreover, the model shows outstanding detection performance in quantitative evaluation metrics, providing strong technical support for early warning and precise control of tomato diseases in practical applications.

## 5 Conclusions and future directions

### 5.1 Conclusion

This research introduces a highly efficient and lightweight object detection approach built on an enhanced version of YOLOv10 for the challenging task of detecting tomato diseases—TomatoGuard-YOLO. By implementing an innovative model architecture and conducting thorough experimental evaluations, notable outcomes have been achieved. The key conclusions are as follows:

1. Incorporating the Multi-Path Inverted Residual Unit (MPIRU) greatly strengthens the model’s capacity for multi-scale feature extraction and integration. Experimental findings demonstrate that MPIRU not only boosts detection accuracy but also lowers model complexity, ensuring effective lightweight detection.
2. The Dynamic Focusing Attention Framework (DFAF) improves the model’s precision in identifying critical disease regions. With the C2f module optimized, C2f-DFAF efficiently captures disease features in complex environments, significantly enhancing detection performance with negligible added computational cost.
3. The Focal-EIoU loss function effectively tackles challenges related to sample imbalance and bounding box regression accuracy, leading to significant improvements in detecting small objects and boundary diseases. In addition to optimizing detection precision, the overall model performance is further improved.

Experimental results indicate that TomatoGuard-YOLO achieves better performance than current methods on the tomato disease dataset. The model achieves an mAP50 of 94.23%, an improvement of 10.07 percentage points over the original YOLOv10, with a model size reduction of 52.76%, parameter count reduction of 65.31%, and an average inference speed increase to 129.64 FPS. These data fully demonstrate the method’s outstanding advantages in model accuracy, efficiency, and lightweight design.

Comparative experiments and visualization results further validate TomatoGuard-YOLO’s excellent performance in complex scenarios. Whether in small target detection, differentiation in complex backgrounds, multi-category disease recognition, or bounding box precision control, the proposed model shows significant improvement. These improvements not only provide reliable technical support for disease detection but also lay a solid foundation for applications in practical agricultural scenarios.

In summary, the TomatoGuard-YOLO model demonstrates excellent performance and broad adaptability in tomato disease detection tasks. Its high accuracy and reliability in disease detection provide powerful technical tools for disease prevention and intelligent management in tomato cultivation, offering significant application value in improving tomato cultivation efficiency and reducing disease losses.

### 5.2 Research limitations and future directions

Despite the significant achievements of TomatoGuard-YOLO in tomato disease detection, the current study lacks large-scale field deployment validation, which is crucial for understanding real-world performance. Future research can further explore the following aspects:

1. Further optimize the design of MPIRU and C2f-DFAF modules, deeply study more efficient feature extraction and attention mechanisms.
2. Another important research direction is to expand the application scope of the model, exploring its generalization ability in detecting diseases of different crops, and testing its detection effectiveness in more complex field scenarios to evaluate its robustness and adaptability.
3. Combining edge computing technology, research on deployment optimization strategies for the model on resource-constrained devices is also key to future development. By optimizing deployment on low-power devices, computational resource consumption can be effectively reduced, further enhancing the model’s practical application value.
4. TomatoGuard-YOLO can work with other agricultural intelligent systems (drone, IoT devices, etc.), fully leveraging the complementary advantages of multi-dimensional data to achieve more comprehensive crop health management in actual agricultural environments. This integration can effectively improve early warning capabilities for diseases, promoting the development of intelligent and precise agriculture.

## Data Availability

The raw data supporting the conclusions of this article will be made available by the authors, without undue reservation.
